# Large-scale in-silico identification of a tumor-specific antigen pool for targeted immunotherapy in triple-negative breast cancer

**DOI:** 10.18632/oncotarget.26808

**Published:** 2019-04-02

**Authors:** Jessica Kaufmann, Nicolas Wentzensen, Titus J. Brinker, Niels Grabe

**Affiliations:** ^1^ Hamamatsu Tissue Imaging and Analysis Center (TIGA), BIOQUANT, University of Heidelberg, Heidelberg, Germany; ^2^ Medical Oncology Department, Universitätsklinik Heidelberg, National Center for Tumor Diseases (NCT), Heidelberg, Germany; ^3^ National Cancer Institute, Division of Cancer Epidemiology & Genetics, Clinical Genetics Branch, NCI Shady Grove, Bethesda, Maryland, USA; ^4^ National Center for Tumor Diseases (NCT), German Cancer Research Center (DKFZ), Heidelberg, Germany; ^5^ Department of Dermatology, University Hospital Heidelberg, Heidelberg, Germany

**Keywords:** immunotherapy, RNA-seq, triple negative breast cancer, target identification, TCGA

## Abstract

Since the advent of cetuximab, clinical cancer treatment has evolved from the standard, relatively nonspecific chemo- and radiotherapy with significant cytotoxic side effects towards immunotherapeutic approaches with selective, target-mechanism-based effects. Antibody therapies as the most successful form of cancer immunotherapy led to approved treatments for specific cancer types with increased patient survival. Thus, the identification of tumor antigens with high immunogenicity is in central focus now. In this study, we applied computational methods to comprehensively discover overexpressed molecular targets with high therapeutic relevance for clinical, immunotherapeutic cancer treatment in triple-negative breast cancer (TNBC). By actively modeling potential negative side effects utilizing expression data of 29 different, normal human tissues, we were able to develop a highly-specific coverage of TNBC patients with RNA targets. We identified here more than 400 potential tumor-specific antigens suitable for targeted therapy, including several already identified as potential targets for TNBC and other solid tumors. A specific cocktail of MAGEB4, CT83, TLX3, ACTL8, PRDM13 achieved almost 94% patient coverage in TNBC. Overall, these results show that our approach can identify and prioritize TNBC targets suitable for targeted therapy. Therefore, our method has the potential to lead to new and more effective immunotherapeutic cancer treatment.

## INTRODUCTION

In the last decade, immunotherapy has emerged as a promising approach for cancer treatment. Immuno-therapeutic strategies against cancer include various approaches. These are ranging from counteracting inhibitory and suppressive mechanisms to stimulating effector mechanisms [1]. Cancer vaccination with tumor antigens as one therapeutic strategy leads to an increase of the ability of the patient's own immune system to leverage an immune response against cancerous cells [2, 3]. Additional strategies encompass adoptive transfer of ex vivo activated T or natural killing cells mediating tumor cell eradication and the use of monoclonal antibodies manipulating tumor-related signaling or stimulating anti-tumor immune response to supply co-stimulatory signals to enhance T cell activity [4–6]. However, substantially increasing the effectivity of immunotherapy in clinical routine, will require the use of appropriate target antigens. Therefore, the choice of therapeutic targets is a critical factor [7].

Ideally, immunotherapeutic strategies specifically target tumors while preserving normal tissues. Nevertheless, far most identified tumor antigens are, at least to some degree, also expressed by normal, healthy tissues leading to immune-related adverse events, inducing hyper-activated T-cell response directed against normal, healthy tissue [8]. Thereby, normal tissue can be differentiated into essential normal tissue (e.g. brain, heart and lung) and non-essential normal tissues. Especially for essential tissues it is crucial to avoid cross-reactivity with the candidate therapeutic molecules. Other healthy tissues or cell populations may be affected without increased morbidity [9]. Therefore, the aim of target selection strategies is to maximize the impact on cancerous cells while avoiding toxicities in essential and minimizing in non-essential normal tissues [8, 10]. Targets fulfilling these requirements are considered highly tumor specific [11, 12].

To identify the highest possible amount of suitable candidate target antigens and determine the optimal balance between sensitivity and specificity, a statistically solid data basis is prerequisite. Building a digital cohort containing as many samples as possible is beneficial for achieving this goal [13]. Particularly important in this regard is compensation of unwanted variations caused by technical and biological biases [14]. In the last decade, several strategies have been proposed to correct phenotypic variation within and between samples [14–16]. Given the homogenized data, meta-analysis across multiple data source can result in an increased statistical power and a decreased bias [13].

Selection of candidate target antigens, overexpressed or expressed exclusively in tumor cells, usually starts with large-scale screening of mRNA enabled by next-generation RNA sequencing [13, 17]. In combination with reference-based alignment strategy, RNA sequencing (RNA-seq) allows a very high level of sensitivity and accuracy leading to revelation of the complex landscape and dynamics of the human transcriptome [18]. Typically, the analysis of RNA-seq data starts with reads being mapped to the genome or transcriptome followed by the assembly of mapped reads into gene-level, exon-level or transcriptome-level expression summaries and normalization of summarized data. As mentioned earlier, especially normalization has been proven to be essential prerequisite in the analysis of RNA-seq data enabling accurate comparison of expression levels between and within samples. Last step for identification of candidate targets is statistical testing of differential expression or absolute comparison of expression levels [19]. As the number of resulting candidate target antigens often is very high and *in vitro* or *in vivo* validation is expensive, prioritization is urgently needed to determine the most promising ones [8]. Various approaches with different criteria for ranking cancer antigens are used. These approaches include prioritizing based on analysis of literature and patents, molecular pathways, cellular location of expression, and clinical databases [20–22].

In this study, we applied a multistage process for identification and prioritization of candidate antigens for targeted therapy in triple-negative breast cancer (TNBC). In general, breast cancer is the most common cancer in women a heterogeneous disease composed of different subtypes [23, 24]. It is categorized in three basic groups depending on the expression of estrogen receptor (ER), progesterone receptor (PR), and human epidermal growth factor receptor 2 (HER2) [23]. The group of TNBCs are defined by the absence of ER, PR and HER2 and constitute 10%-20% of all breast cancers. Triple-negative breast cancer patients are associated with a higher rate of distant recurrence and a poorer prognosis than other subtypes of breast cancer [25, 26]. Unlike other subtypes, cytotoxic chemotherapy is the only systematic treatment option as TNBC are currently lacking any molecular target [23]. Our analysis was based on 98 TNBC samples derived from more than 1000 breast cancer samples by receptor status evaluation from immunohistochemistry (IHC) data. For in-silico identification of overexpressed candidate target antigens in TNBC, cancer gene expression was compared to gene expression in 300 normal samples from 29 different tissues. To systematically identify tumor-specific antigens we developed a multi-stage process generating a candidate target pool for various immunotherapeutic strategies such as vaccination, antibody therapy and adoptive T-cell therapy. Thereby, we prioritized candidate target antigens by combining two key factors for indicating a promising target: specificity and number of patients with antigen-positive cancers.

## RESULTS

**Figure 1:**
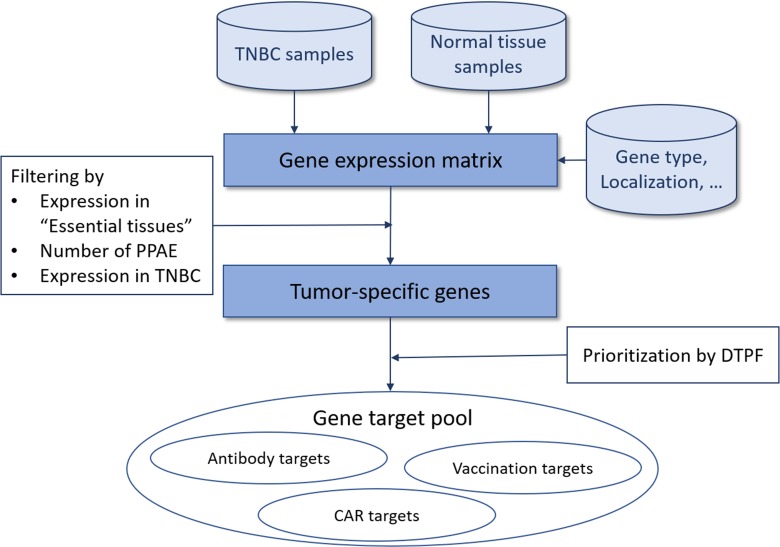
Overview of our approach for tumor-specific antigen selection and prioritization

Our aim was to create a computational strategy for systematic identification and prioritization of *tumor-specific antigens (TSAs)* for targeted therapy. Therefore, we developed a multistage process schematized in Figure 1. In brief, based on the gene expression matrix including normalized expression values for cancer and normal tissue samples, we filtered all protein-coding, overexpressed genes using the gene type information from the Ensembl genome browser in the first step. Next, we identified genes with zero or near-zero expression in “essential" normal tissues, i.e. brain, heart and lung. It is common knowledge that damage to these tissues is life threatening and thus, avoidance of adverse events in these tissues is a fundamental requirement for potential targets. For this goal we classified the expression of all genes of all tissues into *highly*-expressed, *low*-expressed or *non*-expressed, depending on a quantitative mRNA expression threshold determined through multiparametric optimization (see Materials and Methods). Here, we conceptually define a *predicted potential adverse events (PPAE)* as an abstract computational concept of an antigen being *low* or *highly* expressed in any normal tissue. For obtaining suitable, cancer-specific antigens, we then limited the number of PPAE to a maximum of six. We then prioritized the resulting pool of potential cancer-specific antigens based on our *digital target prioritization factor (DTPF)* calculation as described in Material and Methods section.

### Target identification and prioritization

**Figure 2:**
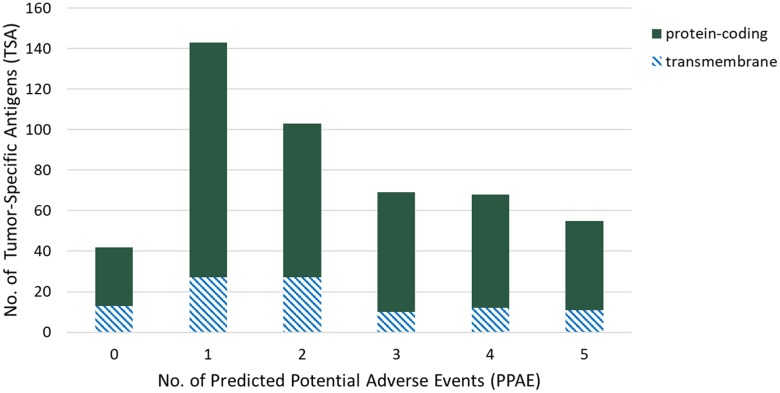
Distribution of tumor-specific antigens (TSAs, y-axis) of predicted potential adverse events (PPAEs, x-axis) and classes of target types

To select candidate TSAs in the first step, we performed gene expression analysis in triple-negative breast cancer samples versus 29 of normal organs and tissues. By filtering genes with *high* expression in at least one tumor sample, *non*-expressed in “essential" normal tissues, i.e. brain, heart and lung, and a maximum number of six PPAEs *(low* or *high* expression) in “non-essential" normal tissues, we identified 480 candidate TSAs, suitable for targeted therapy. Figure 2 shows the distribution of those 480 TSAs over different numbers of predicted potential adverse events (PPAE). With a total number of 143 and 103, most of the candidate TSAs have one or two PPAEs, respectively. For both of these two values of adverse events, 27 candidate TSAs are potentially suitable for an antibody therapy, as the genes are classified as transmembrane with a possible extracellular epitope.

Although, the displayed distribution of candidate antigens over the predicted potential adverse events and classes of targets types gives a good overview of the identified targets, it does not already lead to a prioritization of antigens. Firstly, the “clinical importance" of different tissues has to be taken into account. Depending on the individual tissue or organ affected, an adverse event could have less or more serious implications. Secondly, the relative coverage of a cancer entity by a candidate TSA plays a key role as an ideal target should address as many cancer patients as possible. In order to take both aspects into consideration for target prioritization, we introduced two indices: a predicted potential adverse events (PPAE) index (I_PPAE_) and a tumor sample coverage ratio (TSCR) index (I_TSCR_).

**Figure 3:**
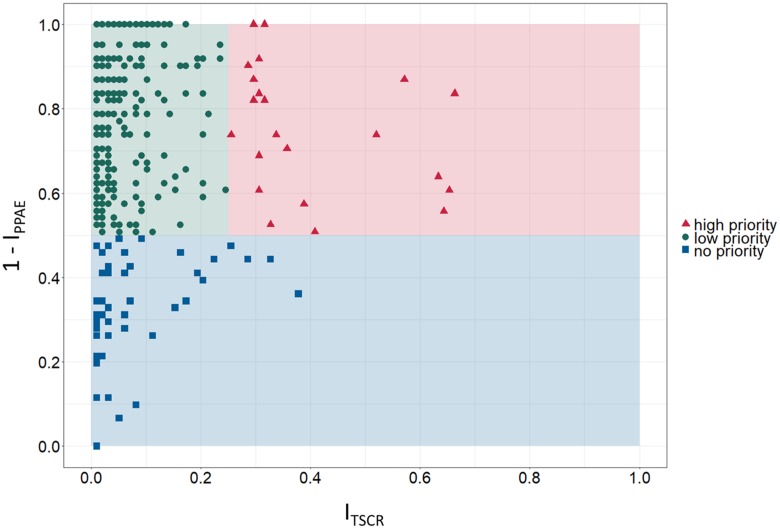
Prioritization matrix for identified tumor-specific antigens (TSA) Exploratory categorization of tumor-specific antigens into high, low and no priority targets based on tumor sample coverage rate index I_TSCR_ (TSCR, x-axis) and 1 - predicted potential adverse events index I_PPAE_ (1 - PPAE, y-axis).

For prioritization of identified candidate TSAs, PPAE index (I_PPAE_) as well as TSCR index (I_TSCR_) were calculated as described in the Materials and Methods section. Figure 3 shows the scatter graph to rank the identified cancer-specific antigens based on determined I_TSCR_ (x-axis) and 1 - I_PPAE_ (y-axis). The spatial positions in the scatter graph was used to roughly classify a candidate TSA into one of three classes: high priority, low priority, and no priority. Exploratory thresholds for classification were set to 0.5 and 0.25 for 1 - I_PPAE_ and I_TSCR_, respectively. An ideal target for immunotherapy has as less adverse events as possible and can, at the same time, addresses as many patients as possible. Therefore, a TSA was considered as high priority if the 1 - I_PPAE_ was higher than the defined threshold of 0.5 and I_TSCR_ was higher than the defined threshold of 0.25. If the 1 - I_PPAE_ was higher than 0.5, but I_TSCR_ was less than 0.25 a candidate TSA was considered as low priority. Accordingly, a TSA with a 1 - I_PPAE_ less or equal than the threshold of 0.5 and either less or equal than or greater than the I_TSCR_ threshold 0.25 was classified as candidate TSA with no priority. Considering these boundaries for classification, 23 candidate TSAs are classified as targets with high priority, 408 TSAs as targets with low priority and the remaining 49 as targets with no priority. Given these numbers, most of the identified TSAs were classified as low priority.

Based on the definition of an ideal, high priority target – avoidance of adverse events in normal tissues and organs and high coverage of cancer patients – Figure 3 shows 23 candidate TSAs according to that definition. Furthermore, 6 of these 23 TSAs are expressed in more than 50% of the examined samples, 3 of them even with 1 - I_PPAE_ greater than 0.7. Additionally, 2 other potentials cancer-specific antigens classified as high priority targets have a smaller proportion of addressable patients (~30%) but have a 1 - I_PPAE_ of 1.0.

The 408 potential TSAs classified as low priority targets are likely not the very first choice for immunotherapy as they are covering a smaller proportion of breast cancer patients but are nevertheless suitable as targets. Especially considered in rather personalized immunotherapy these TSAs can act as candidate targets as they still have a low PPAE index.

Only 49 of the total amount of 480 identified TSAs do not get any priority as they violate both criteria of an ideal target. Although these genes are not present in the essential tissues brain, heart and lung, the probability of potential adverse events is too high. For safety reasons, the 49 identified TSAs were therefore excluded for further analyses.

Depending on chosen thresholds, the number of TSAs with high, low, or no priority are varying. In order to avoid such a strict categorization, we introduced a new qualitative score. By combining I_PPAE_ and I_TSCR_, we developed the digital target prioritization factor (DTPF), allowing a fixed order of identified TSAs.

For the 480 potential TSAs identified in the first step, genes were ordered according to digital target prioritization factor (DTPF). The prioritized top-15 protein-coding TSAs are listed in Table 1. Each gene is listed along with the average expression value in tumor samples, the number of TNBC samples in which the gene is highly expressed (coverage), the number of PPAEs and the DTPF. The calculated DTPF ranges from 1.499 for tumor-specific antigen CT83 (ranked 1^st^) to 1.153 for DMRT1 (ranked 15^th^). The coverage for the top-15 TSAs range from 17 TNBC samples for C4orf51 (ranked 13^th^) up to 65 TNBC samples for CT83 (ranked 1^st^). The highest average expression values, calculated across all cancer samples, have candidate tumor-specific antigens ACTL8 (ranked 2^nd^), MAGEA6 (ranked 14^th^) and C6orf15 (ranked 8^th^) with a normalized count expression of 2167.8, 981.4 and 787.0 respectively. Most of the top-15 identified TSAs have two PPAEs (7 targets), including the top-2 ranked genes CT83 and ACTL8 in the list. Five of the top-15 candidate TSAs have even less than two PPAEs (MAGEB4, TLX3, PRDM13, ERVV-2, and C4orf51). Only three of the top-15 list have more than three PPAEs (OBP2B (5), CLPSL1 (5), and MIA (4)).

**Table 1:** Top 15 tumor-specific antigens (TSA) prioritized by digital target prioritization factor (DTPF)

	Gene	Avg. Expression	TSCR	PPAE	DTPF
**1**	CT83	561	0.66	2	1.499
**2**	ACTL8	2168	0.57	2	1.440
**3**	MAGEB4	86	0.32	1	1.316
**4**	TLX3	88	0.32	0	1.316
**5**	PRDM13	71	0.30	1	1.296
**6**	OBP2B	732	0.63	5	1.272
**7**	CLPSL1	277	0.65	5	1.260
**8**	C6orf15	787	0.52	2	1.258
**9**	DMBX1	94	0.31	2	1.224
**10**	MIA	256	0.64	4	1.200
**11**	ERVV-2	61	0.29	1	1.187
**12**	C4orf51	44	0.17	1	1.173
**13**	MAGEA6	981	0.30	2	1.165
**14**	DMRT1	53	0.23	2	1.153
**15**	OR2B6	63	0.31	2	1.142

Based on the ranked list of tumor-specific antigens, we performed further characterization of top targets by evaluating expression profiles. Especially potentially appearing adverse events based on the number of PPAE were further investigated.

**Figure 4:**
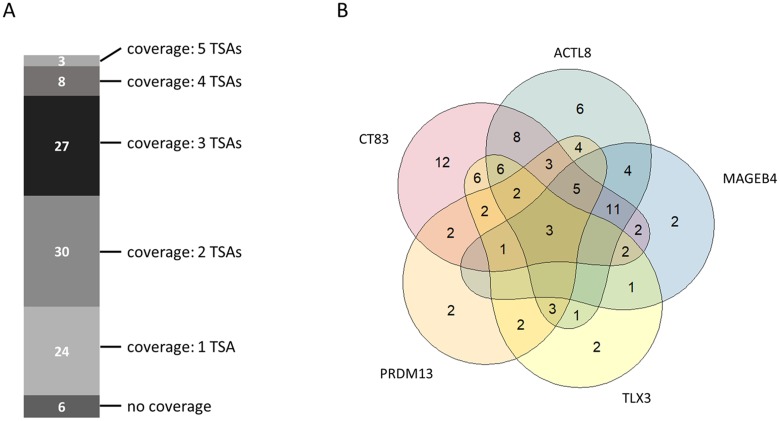
Collective tumor sample coverage for the identified top 5 tumor-specific antigens (TSAs) **(A)** Multi-coverage of tumor samples by identified top 5 tumor-specific antigens. **(B)** Venn-Diagram showing the possible number of tumor samples covered by a single tumor-specific antigen or by a combination of the identified top 5 tumor-specific antigens.

Beside the importance of tumor sample coverage rates for single tumor-specific antigens, collective coverage of tumor-specific antigen combinations was of great interest for us. Analyzing the coverage for combinations of tumor-specific antigen allows to ascertain the number of tumor patients benefitting from a combinatorial immunotherapy comprising multiple therapeutic molecules. Therefore, we investigated the collective coverage of top 5 ranked tumor-specific antigens. Figure 4A shows the multi-coverage of tumor samples by the top 5 ranked tumor-specific antigens. Out of 98 tumor samples, 24 samples (~24.5%) are covered by one, 30 samples (~30.6%) are covered by two, 27 samples (~27.6%) are covered by three, 8 samples (~8.2%) are covered by four, and 3 samples (~3.1%) are covered by all five of the top 5 ranked TSAs. Only 6 out of the 98 analyzed tumor samples are not covered by the top 5 ranked TSAs. Therefore, the total coverage for the top 5 ranked TSAs is 93.9%.

Figure 4B depicts the tumor sample coverage of all possible combinations of top 5 ranked tumor-specific antigens. CT83 (ranked 1^st^) already covers 65 out of 98 tumor samples (~66.3%). In combination with ACTL8 (ranked 2^nd^), the collective coverage is increased to 83 out of 98 tumor samples (84.7%). Adding MAGEB4 (ranked 3^rd^) as third therapeutic molecule increases the collective coverage to 87.8%.

Based on the ranked list of tumor-specific antigens and the analysis of collective coverage, we performed further characterization of top targets by evaluating expression profiles. Especially potentially appearing adverse events based on the number of PPAE were further investigated.

### Top target characterization

**Figure 5:**
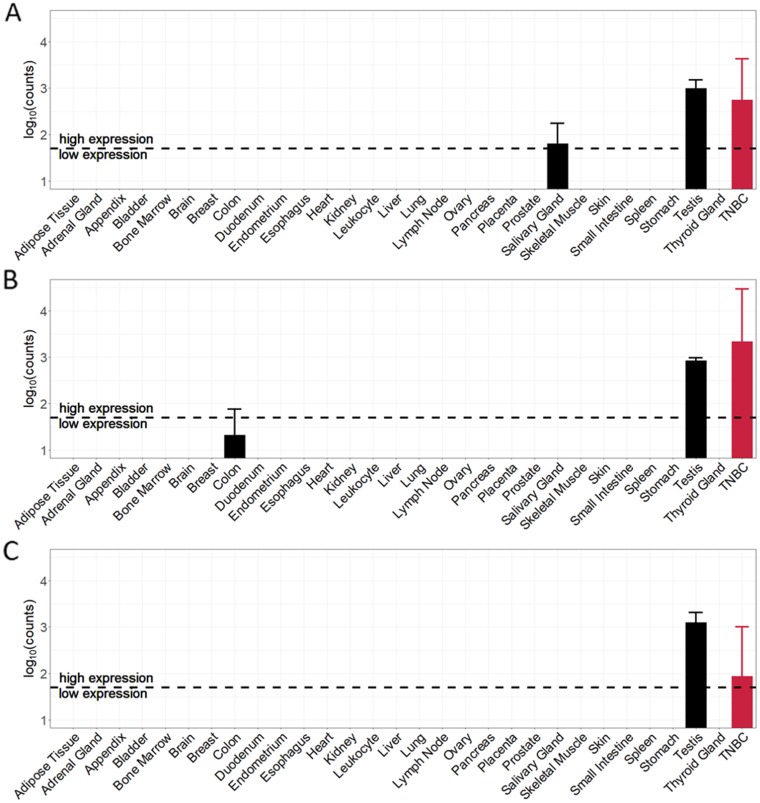
Cancer and normal tissue expression profile of the top-3 tumor-specific antigens (TSA) identified for TNBC samples **(A)** CT83 (ranked 1^st^). **(B)** ACTL8 (ranked 2^nd^). **(C)** MAGEB4 (ranked 3^rd^).

Expression values of the top-3 TSAs in each normal tissue sample and triple-negative breast cancer sample are shown in Figure 5. The expression of tumor-specific antigens in TNBC samples is very high compared to the expression in almost all normal tissue samples. The expression profile of CT83 (Figure 5A) shows high average expression values for two normal tissues – salivary gland (62.4) and testis (988.8) and an average expression in TNBC samples of around 561 counts. Tumor-specific antigen ACTL8 (Figure 5B) shows high average expression values in testis (848.1) and low expression in colon (20.2). The average expression in triple-negative breast cancer samples with a value of around 2168 counts is the highest average value of all top-15 candidate TSAs. The 3^rd^ ranked antigen MAGEB4 (Figure 5C) has only one PPAE in testis tissue with an average expression (1252.1). The normalized average expression in the TNBC samples for MAGEB4 is around 86 counts.

Given that triple-negative breast cancer patients are female, we here neglected the predicted potential adverse events in testis tissue. Regarding the top-3 tumor-specific antigens, remaining PPAEs are salivary gland for targeting CT83 and colon for targeting ACTL8.

After identifying and characterizing the ranked list of TSAs, we further investigated applicability of top TSAs for vaccination and adoptive T-cell therapies. Therefore, we determined 9-mer peptides originating from identified TSAs most likely forming peptide-HLA complexes.

**Figure 6:**
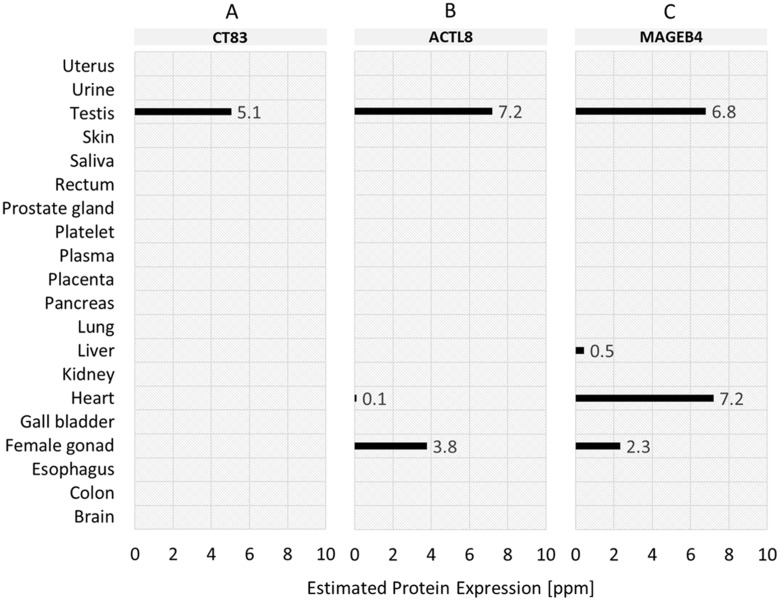
Normal tissue protein expression profiles of the top-3 tumor-specific antigens (TSA) identified for TNBC samples **(A)** CT83 (ranked 1^st^). **(B)** ACTL8 (ranked 2^nd^). **(C)** MAGEB4 (ranked 3^rd^).

Based on the analysis of mRNA expression, we furthermore investigated whether the normal tissue expression profiles also apply on protein level. The protein expression values of the top-3 TSAs in normal tissues are shown in Figure 6. The protein expression profile of CT83 (Figure 6A) confirms the analysis of mRNA expression for testis – expression value 5.1 ppm. For salivary gland, in contrast, no expression on protein level is specified. Protein expression profile for ACTL8 (Figure 6B) shows – in accordance with mRNA expression analysis – a protein expression value in testis (7.2 ppm). In contrast to mRNA expression, ACTL8 is additionally low expressed in heart (0.1 ppm) and moderately expressed in female gonad (3.8 ppm). For the 3^rd^ ranked antigen MAGEB4 (Figure 6C) the mRNA expression in testis is also specified on protein expression level (6.8 ppm). Additionally, MAGEB4 is – in contrast to mRNA expression – also expressed in liver (0.5 ppm), heart (7.2 ppm) and female gonad (2.3 ppm) on protein level.

The top-15 TSAs that in complex with HLA-A^*^02:01 allele form the top-40 cancer-specific 9-mer peptide-HLA targets in context of affinity (IC50) are listed in Table 2. Each peptide is listed along with the gene from which the peptide was identified, the predicted binding affinity and the classification of peptides in strong and weak binders, derived from the predicted binding affinity. 38 of the top-40 peptides have an IC50 values of less than 50nM and are therefore classified as strong binders, indicating that peptide and HLA molecule are binding very tightly together. The IC50 values of top-11 peptides in the list are even smaller than 10nM. The strongest binding affinity we determined was given at peptides KMLEILFEL (ATCL8, 2.18 nM), LLAWAISPV (MIA, 3.95 nM), and ALPSLNWFV (ERVV-2, 4.43 nM). For the candidate gene ERVV-2, the highest number within the top-40 of possible 9mer peptides binding with high affinity to HLA-A^*^02:01 was identified. In total 10 potential peptides for ERVV-2 were predicted. For the top-3 candidate targets CT83, ACTL8, and MAGEB4 described in previous sections, 4, 1 and 3 potential 9mer peptides within the top-40 peptides have been predicted.

**Table 2:** Top-40 potential peptide-HLA complexes for top-15 candidate TSAs binding to HLA-A^*^02

	Gene	Peptide	IC50 (nM)	Strong/weak binders
**1**	ACTL8	KMLEILFEL	2.18	strong binder
**2**	MIA	LLAWAISPV	3.95	strong binder
**3**	ERVV-2	ALPSLNWFV	4.43	strong binder
**4**	MAGEB4	KVLEFLAKV	6.26	strong binder
**5**	ERVV-2	FLFLYLSLL	6.64	strong binder
**6**	CLPSL1	LLFFFLFLL	8.2	strong binder
**7**	DMBX1	RLADIILEA	8.34	strong binder
**8**	ERVV-2	YLSLLPMPL	8.94	strong binder
**9**	DMRT1	SLFPYYNNL	9.04	strong binder
**10**	C6orf15	GLFARSIGV	9.14	strong binder
**11**	CLPSL1	FLLFFFLFL	9.29	strong binder
**12**	ERVV-2	SLNWFVPLL	10.3	strong binder
**13**	MAGEB4	LLMPLLSVI	10.69	strong binder
**14**	DMRT1	MVIQDIPAV	12.04	strong binder
**15**	CT83	KLVELEHTL	12.24	strong binder
**16**	CLPSL1	LLFLLFFFL	14.63	strong binder
**17**	MIA	YMAPDCRFL	14.71	strong binder
**18**	C6orf15	ALPEELSYL	14.79	strong binder
**19**	CLPSL1	MMLPQWLLL	17.12	strong binder
**20**	CLPSL1	MLPQWLLLL	17.88	strong binder
**21**	ERVV-2	YLLAEQGGV	18.37	strong binder
**22**	MAGEB4	GLLMPLLSV	20.03	strong binder
**23**	ERVV-2	FLYLSLLPM	20.03	strong binder
**24**	C6orf15	YLSSAAALA	20.58	strong binder
**25**	MAGEA6	YIFATCLGL	20.58	strong binder
**26**	ERVV-2	YILVRNFSL	25.27	strong binder
**27**	DMBX1	YLGVNMAPL	25.69	strong binder
**28**	PRDM13	KLYSRKYGL	28.01	strong binder
**29**	ERVV-2	SLANSAHQV	28.78	strong binder
**30**	DMBX1	GLAPASATL	32.24	strong binder
**31**	ERVV-2	KITYSTPPV	32.41	strong binder
**32**	CT83	ILNNFPHSI	32.94	strong binder
**33**	MAGEA6	FLWGPRALI	36.31	strong binder
**34**	CT83	YLLLASSIL	38.12	strong binder
**35**	C4orf51	ILLPFSPLT	42.94	strong binder
**36**	MAGEA6	KIWEELSVL	45.09	strong binder
**37**	PRDM13	GLLKYPESI	46.57	strong binder
**38**	ERVV-2	NLYTCINNI	49.16	strong binder
**39**	DMBX1	HMAATNNLV	52.74	weak binder
**40**	CT83	LLASSILCA	53.9	weak binder

## DISCUSSION

In this paper, we have presented a unique approach for selecting and prioritizing tumor-specific antigens serving as potential targets in immunotherapeutic strategies. The strength of our strategy is the parameterized identification and prioritization of candidate targets adaptable to different requirements and resulting in a pool of tumor-specific antigens usable for various kinds of targeted immunotherapy. After a comprehensive analysis of 98 triple-negative breast cancer samples together with 345 normal tissue samples from 29 different tissue types, we were able to identify more than 400 candidate targets. All identified candidate targets had zero or near zero expression (*non*-expression category) in defined “essential" normal tissues brain, heart and lung and had less than six predicted potential adverse events in defined “non-essential" normal tissues. Due to our introduced digital target prioritization factor, we were able to rank the identified candidate targets and to focus further investigations on the most promising ones. The top ranked candidate targets were characterized by both, minimal effect on normal tissues, as well as maximal number of patients potentially benefitting of an immunotherapy. Further evaluation of top ranked candidates showed that 38 cancer-specific 9mer peptide-HLA complexes are predicted to bind very tightly together and are therefore promising targets for either tumor vaccination or adoptive T-cell transfer.

Of the identified, cancer-specific antigens in our list, the cancer/testis antigen 83 (CT83) had the most interesting profiles with high expression in more than 65% of triple-negative tumor samples and lower expression in almost all normal tissues examined. To our knowledge, CT83 is absent from current immunotherapy development, but has already been identified as a potential target in triple-negative breast cancer [27] and lung adenocarcinoma [28]. Like CT83, also Actin like 8 (ACTL8) is already described as a potential target in breast cancer [29, 30] and is, to our knowledge, absent from current immunotherapy development as well. ACTL8 is highly expressed in 57% of the analyzed triple-negative breast cancer samples and potential side effects on an immunotherapy is only predicted for colon with a very low expression level.

The expression of MAGEB4 reported by PaxDB contradicts the negative RNA expression as reported by the Human Protein Atlas (HPA) [31], the GTEx [32] and FANTOM5 dataset [33] and the protein expression as reported by HPA. MAGEB4 is a well-known Cancer Testis Antigen (CTA) [34]. Its absence of expression in heart is also in line with the proposed functional role of MAGE proteins in developmental processes and tumor emergence [35]. This renders the specific result in heart tissue from PaxDB a potential false positive unless further evidence is shown.

Although the approach described here is focused on triple-negative breast cancer, with some modifications it can be used for different cancer types. As it is designed as parameterizable framework, adaptions on the expression level of “essential" tissues, the number of predicted potential adverse events (PPAEs) or the tissue weights for the digital target prioritization factor (DTPF) can easily be applied.

We are aware that our strategy, in line of the proposed thinking, can further be refined, e.g. towards the potential cross-reactivity of peptides. A peptide that has 5 to 8 identical amino acids can potentially lead to off-target effects as it might have a peptide that is similar to the identified target. Therefore, it is important to not only investigate whether a tumor-specific antigen is specifically expressed in cancer cells and not in normal tissues. Furthermore, also a peptide-HLA complex has to meet these conditions. Future work could address this issue by identification of similar peptides in the human proteome and calculation of degree of similarity for each of the peptides in the potential cancer-specific complexes as described in [36]. Second, we here only studied to 9-mer peptides and HLA-A^*^02:01. This could further also be expanded e.g. to variable length of peptides and different HLA alleles for peptide-HLA complex prediction. Interesting would also be the prediction of epitope length for transmembrane candidate targets. Another refinement could also address a more detailed automatic characterization of the extracellular domain of the transmembrane proteins identified.

As a disclaimer, it needs to be emphasized that the identified candidate targets are based purely on in-silico work and therefore require experimental validation which is beyond the scope of this paper. Given the limitations described above, a subset of the targets may not be valid.

## MATERIALS AND METHODS

### RNA-seq data collection and gene expression calculation

Raw RNA sequencing data was obtained from the TCGA (The Cancer Genome Atlas) (TCGA-BRCA, TCGA-PRAD, TCGA-LUAD) and the ArrayExpress (E-MTAB-1733, E-MTAB-513) [37]. The data corresponded to 443 samples in total. 98 of 443 samples were triple-negative breast cancer samples from TCGA-BRCA identified by TCGA Barcode and corresponding clinical data about receptor status of ER, PR and HER2. In case of equivocal HER2 receptor status results of FISH test were considered. 345 of 443 samples were normal, tumor-free samples from 29 different tissue types (adipose tissue, adrenal gland, appendix, bladder, bone marrow, brain, breast, colon, duodenum, endometrium, esophagus, heart, kidney, leukocyte, liver, lung, lymph node, ovary, pancreas, placenta, prostate, salivary gland, skeletal muscle, skin, small intestine, spleen, stomach, testis, thyroid gland). To exclude any potential discrepancy, a common data processing pipeline was used. Therefore, alignment of the raw data against human reference genome GRCh38 from Genome Reference Consortium (GRC) was performed using STAR version 2.4.0e [38]. The reads mapped to each gene were enumerated using HT-Seq count version 0.6.1 [39]. GENCODE v21 [40] was used for gene annotation. Data analysis on the expression values provided by HTSeq count was performed using R version 3.3.3 [41] and Bioconductor version 3.4 [42]. For data normalization the R package DESeq version 1.26.0 [43] was used. Afterwards, normalized count expression data was stored in a MySQL database and enriched with gene type and subcellular localization information from Ensemble [44]. In preparation for target identification, the average count value of each gene per normal tissue type was derived.

### Target identification

Identification of the largest possible tumor-specific antigen pool was performed by maximization of three targets: absolute number of TSAs, average number of TSAs per sample, and average number of samples per TSAs. Key determinants for target maximization were tumor expression (read counts), expression in “essential" normal tissues (read counts), i.e. brain, heart and lung, and number of predicted potential adverse events (PPAE) in “non-essential" normal tissues (definition above). Maximization of the three target figures was done using multiparametric optimization based on gradient ascent. For this we defined a potential parameter field. In this field we assumed PPAEs for a tumor-specific antigen and normal tissue to have the average gene expression for that antigen of bigger than 10 read counts in the respective normal tissue type. The examined value range for tumor expression was between 10 and 100 read counts. For “essential" normal tissue expression was studied between 5 and 20 read counts, and for the number of predicted potential adverse events “non-essential" normal tissues a range between 0 and 8 was studied. Multiparametric optimization then showed that potential targets were those with more than 50 read counts in at least one TNBC sample, less than on average 10 read counts in “essential" tissues (brain, heart and lung) and less than six predicted potential adverse events in “non-essential" normal tissues.

### Target prioritization

For prioritizing the identified tumor-specific antigens, two quantitative indices were calculated and combined into the digital target prioritization factor (DTPF): predicted potential adverse event index (I_PPAE_) and tumor sample coverage ratio index (I_TSCR_). Inspired by the Sequential Organ Failure Assessment (SOFA) score used to evaluate the condition of patients in Intensive Care Units (ICU), the introduced PPAE index (I_PPAE_) included weighting tissues for quantitatively reflecting a generalized clinical importance of different tissue types within the human body. For each tumor-specific antigen *a*, the predicted potential adverse event index I_PPAE_(*a*) was calculated as follows:

$$I_{PPAE}\left(a\right)=\;\frac{\sum_{t=1}^{T}e\left(a,t\right)*w(t)}{\max(\sum_{t=1}^{T}e\left(a,t\right)*w(t))}$$

where *t* is the tissue index in the given amount of T tissue types. e(*a,t*) is the expression indicator for an identified tumor-specific antigen a in normal tissue type t, whereby e(a,t) ϵ {0,0.5,1}. Based on the results in multi-parametric optimization buckets for the expression indicator were defined as follows: None expression (0) is defined as a tumor-specific antigen a that has less or equal than 10 read counts in normal tissue type t, low expression (0.5) is defined as a tumor-specific antigen a that has more than 10 read counts but less or equal than 50 read counts in normal tissue type t, and high expression (1) is defined as a tumor-specific antigen a that has more than 50 read counts in tumor type t. w(t) is the weight for tissue type *t*. The tissue weight values, ranging between 0 and 1, are shown in Table 3.

**Table 3:** Tissue weight values for predicted potential adverse event index (I_PPAE_)

Weight	Tissue types	Clinical relevance
**1**	Bone marrow Kidney Liver Leukocytes	Immediately life threating
**0.8**	Colon Duodenum Esophagus Stomach Adipose tissue Small intestine Pancreas	Life threating
**0.5**	Adrenal gland Bladder Skin Thyroid Salivary gland Skeletal muscle Lymph nodes	Not immediately life threating
**0.3**	Appendix Gall bladder Endometrium Breast Ovary Spleen Placenta	Not life threating
**0**	Testis Prostate	No affect

The tumor sample coverage ratio index I_TSCR_ for a tumor-specific antigen a is calculated as:

$$I_{TSCR}\left(a\right)=\;\frac{\sum_{s=1}^{M}t(a,s)}{M}$$

where *s* is the sample index in the given amount of M triple-negative breast cancer samples. Furthermore, *t(a,s*) is the target indicator for an identified tumor-specific antigen a in tumor sample s. The value of *t(a,s*) is 0 if the expression is less or equal than the defined tumor threshold of 50 counts and 1 if the expression is higher.

The digital target prioritization factor DTPF(*a*) for an identified tumor-specific antigen combined I_PPAE_ and I_TSCR_ as follows:

$$DTPF\left(a\right)=\left(1-I_{PPAE}\left(a\right)\right)+\;I_{TSCR}(a)$$

### Protein expression levels

Protein expression values for top ranked tumor-specific antigens were obtained from PaxDB [45]. PaxDB is a comprehensive absolute protein abundance database, providing integrated datasets, which aggregate and average protein expressions over the various samples, conditions and cell-types resulting in high coverage and data quality. All protein abundances are given in ppm, which is short for parts per million. After identification of tumor-specific antigens with highest DTPF, protein expression data for the top-3 ranked TSAs were selected and downloaded as.tsv file from PaxDB. Afterwards, the downloaded file was filtered only for integrated datasets, resulting in average protein expression values for 20 different normal tissues (brain, colon, esophagus, female gonad, gall bladder, heart, kidney, liver, lung, pancreas, placenta, plasma, platelet, prostate gland, rectum, saliva, skin, testis, urine, uterus).

### Detection of potential peptide-HLA complexes

For tumor-specific antigens with high DTPF, potential peptide-HLA complexes are identified using NetMHCCons webserver 1.1 [46]. NetMHCcons uses an artificial neural network-based (ANN) allele-specific method to predict binding of peptides to any known MHC class I molecule. For a given peptide sequence and an allele name the program predicts the IC50 affinity. In general, the predicted binding affinity estimates how tightly the peptide and the HLA molecule bind to each other. IC50 is defined as a dose of peptides that displaces 50% of a competitive ligand. A peptide is considered a strong binder to a HLA allele, if the IC50 value is smaller than 50 nanomolar (nM) and a weak binder if the IC50 value is smaller than 500 nanomolar (nM). As the HLA-A^*^02 genes are those with highest allelic frequency in European Caucasian population [47], we here evaluated peptides of identified tumor-specific antigens for binding affinity against this allele.

## CONCLUSIONS

Preserving healthy tissue while specifically targeting cancerous cells is a primary objective of cancer immunotherapy. Therefore, a key feature of an “ideal" target is a highest possible expression in cancer cells and no or very low expression in all normal tissue types. Following this fundamental requirement, our multi-stage process provides a unique approach to select and prioritize tumor-specific antigens serving as a candidate target pool for various immunotherapeutic strategies. We applied our strategy to triple-negative breast cancer (TNBC) where patients have a generally poorer prognosis as targeted therapies are currently unavailable. A first in-silico evaluation of prioritized target pool revealed our strategy as a promising starting point which will hopefully lead to develop better immunotherapies with minimal adverse side effects for also all other cancer types.
